# The effect of the Tai Chi intervention on self-esteem and self-confidence perception in adult populations: a systematic review and meta-analysis

**DOI:** 10.1186/s12912-025-02792-9

**Published:** 2025-02-14

**Authors:** Sek Ying CHAIR, Bernard Man Hin LAW, Aileen Wai Kiu CHAN, Ruitong GAO

**Affiliations:** The Nethersole School of Nursing, Faculty of Medicine, The Chinese University of Hong Kong, Shatin, N.T., Hong Kong SAR China

**Keywords:** Tai Ji, Self Concept, Adult, Meta-analysis

## Abstract

**Background:**

Self-esteem is a psychological outcome that is positively related to mental and psychological health, one of the most important elements in nursing. Developing strategies for self-esteem promotion is therefore of great importance in nursing practice. Previous reviews suggested that exercise interventions such as Tai Chi practice may confer both mental and physical benefits in humans, but reviews on the effect of Tai Chi exercise on self-esteem of adults are lacking. Thus, this systematic review and meta-analysis was conducted to evaluate the effectiveness of Tai Chi interventions in promoting self-esteem among adults.

**Methods:**

We included randomized controlled trials that investigated Tai Chi interventions in adults, where self-esteem and/or self-confidence were reported as outcomes. The literature search was conducted in ten electronic databases. Two independent reviewers performed abstract and full-text screening using Covidence. These reviewers also independently extracted data from the included studies, and conducted critical appraisal of their methodological quality, using the revised Cochrane risk-of-bias tool for randomized trials (RoB2).

**Results:**

Eleven studies were included in the review, of which all were rated as having high risk of bias or some concerns in their methodological quality. Our meta-analysis demonstrated that Tai Chi intervention can significantly improve self-esteem among adults, where the post-intervention self-esteem score was higher among the intervention participants compared to the controls (standardized mean difference: 0.46; 95% CI: 0.18–0.74; *p* = 0.001). Sensitivity analyses by excluding studies not reporting data using means and standard deviations revealed similar findings. One study also showed a positive effect of Tai Chi intervention on self-confidence perception.

**Conclusions:**

Tai Chi interventions have a moderately positive effect on self-esteem among adults. Tai Chi exercise classes could be a potential strategy to implement within communities, especially among individuals in higher need to improve their self-esteem such as older adults and patients having chronic illnesses that have detrimental effects on their psychological well-being. However, given the high risk of bias in most studies, caution is advised before recommending widespread implementation. Further high-quality research, including qualitative studies exploring how Tai Chi can improve self-esteem, is needed to strengthen the evidence base.

**Supplementary Information:**

The online version contains supplementary material available at 10.1186/s12912-025-02792-9.

## Introduction

Self-esteem can be considered as a measure on whether a person is able to have “a sense of self-worth” and have a positive attitude towards himself or herself [1]. It is a psychological outcome that was found to be associated with well-being [1], and showing a positive correlation with life satisfaction [2]. Positive self-esteem is viewed as central to human mental health and well-being, which can actively promote healthy functioning of various life aspects like achievements and satisfaction, and the ability to cope with chronic conditions such as cardiovascular diseases and cancer [3]. Conversely, low self-esteem can be associated with the development of negative mental health outcomes, such as depression and anxiety [4, 5]. In addition to a high degree of personal suffering, low self-esteem may cause social problems such as violence, substance abuse and high-risk social behaviors, imposing a substantial burden on society [6]. Therefore, examination and development of strategies that may improve one’s self-esteem is of paramount importance, for the benefit of one’s psychological health and quality of life [7–9].

Notably, promotion of self-esteem is considered to be an important part of the scope of practice in health care, given the importance of self-esteem in the promotion of mental health and outcomes related to the management and recovery of chronic disease [10, 11]. Indeed, the occurrence of certain chronic diseases was found to be causal to a reduction in self-esteem among patients [12]. For example, breast cancer patients were reported to face issues regarding their body image, due to changes in body appearance as a result of breast cancer treatment including hair loss, breast asymmetry and potential weight gain [13, 14]. Such body image issues would in turn lead to a loss of self-esteem among the breast cancer patients and survivors, which would have a detrimental effect on their quality of life [15]. Additionally, these physical and psychological changes would reduce women’s perceived self-efficacy to cope with their cancer symptoms, further exacerbating their well-being [16, 17].

Over the past decades, studies adopting a variety of interventions have demonstrated the significant effects of these interventions in enhancing self-esteem. These include cognitive behavioral therapies, reminiscence-based interventions, physical activity interventions, mindfulness-based interventions and support groups [3, 18, 19]. Nevertheless, these interventions only confer their benefits on self-esteem promotion through either a holistic approach or physical approach alone. Other intervention types that utilize a combination of holistic and physical approaches for self-esteem promotion could be of greater benefits for self-esteem promotion. One such type of intervention is Tai Chi intervention.

Tai Chi is a mild-to-moderate aerobic exercise involving different number of movements or forms, and is considered to be a low-demand and safe option for adults to improve physical fitness including muscle strength, flexibility, postural balance and physical endurance [20]. Through relaxed breathing and mental concentration, the meditative movements of Tai Chi promote healing of both the body and mind, enhancing self-awareness and a sense of inner peace [21]. Tai Chi class is often practiced as a group, which can help promote a sense of togetherness, and in turn enhance social support, self-efficacy and cognitive function [22]. Moreover, Tai Chi interventions were shown to be effective in reducing falls and improving balance among older adults [23], a factor that may improve their self-esteem and confidence [24]. Taken together, Tai Chi can be considered an exercise that confers both mental and physical benefits.

To date, reviews on Tai Chi interventions primarily focus on their effects on outcomes pertaining to disease prevention and physical health such as the occurrence of falls/balance, neurological diseases, cardiovascular diseases, musculoskeletal diseases, cancer, and diabetes [25]. With Tai Chi involving the holistic and physical components that are both known to improve self-esteem, it is conceivable that Tai Chi interventions are an effective strategy for self-esteem promotion. Yet, convincing evidence from systematic reviews to promote self-esteem with Tai Chi are currently lacking, although recent meta-analyses had reported the positive effect of Tai Chi interventions on anxiety and depression [26, 27] and quality of life [28], outcomes that are generally related to self-esteem. To address this research gap, this review summarized the current evidence for the potential effects of Tai Chi on self-esteem among adult populations by critically appraising and synthesizing findings from previously published randomized controlled trials. The synthesized evidence would be of value for informing health policy makers on the benefits of the implementation of Tai Chi exercise interventions as part of the health promotion programs offered at organizations serving the local community.

## Methods

The study is reported following the PRISMA 2020 Statement [29] and has been registered in the international prospective register of systematic reviews (Reference Number. CRD42023439055). The protocol for this meta-analysis was not previously published.

### Search strategy

The following seven electronic databases, PubMed, Embase, Web of Science, CINAHL, the Cochrane Library, PsycINFO and OVID MEDLINE were systematically searched to identify studies from their inception to June 2023. In addition, Chinese databases including China National Knowledge Infrastructure (CNKI), Wanfang, and China Science and Technology Journal Database CQVIP were used to search for relevant articles published in Chinese. As Tai Chi Qi Gong is also a widely used type of Tai Chi, we also included the Qi Gong in the search strategy. Keywords such as “self-concept”, “self-confidence”, “self-esteem” were used for searching self-esteem. The search strategy that was used is shown in Supplementary Table 1. We have also manually checked the reference lists of relevant articles to identify potential publications.

## Inclusion and exclusion criteria

### Participants

Studies involving samples of adult populations aged 18 or above were included.

## Interventions

Studies reporting Tai Chi interventions or Tai Chi Qigong interventions were included. However, studies reporting Qigong interventions alone were excluded.

## Comparisons

Studies comparing the effect of Tai Chi intervention with usual care, routine activities, waiting list control, or other non-exercise standard intervention (including psychosocial support therapies or educational interventions) were included. Studies that compare groups receiving (1) Tai Chi interventions coupled with usual care or (2) usual care alone, were also included.

### Outcomes

Studies reporting outcomes of self-esteem and/or self-confidence perceptions were included.

## Study design

Studies reporting randomized controlled trials were included, while studies utilizing other study designs were excluded.

## Additional criteria

Only studies published in English or Chinese were included, based on the linguistic capability of the author team. Conference abstracts, protocols, reviews, clinical guidelines, comments, letters, editorials, or articles without full texts were excluded.

### Study selection and data extraction

Covidence was used during the selection of articles for inclusion in the review. The retrieved citations from the searched databases were imported into Covidence. After removing the duplicates, the titles and abstracts of the articles were initially screened by two independent reviewers (BMHL, RG) and were selected for further examination of the full-text based on the aforementioned inclusion and exclusion criteria. Thereafter, the full-text of the selected articles were examined to assess their suitability for inclusion in the review. Disagreements between the reviewers on whether a study was to be included in the review were resolved through discussions.

Data extracted from the included studies include participant characteristics (population, sample size, age, gender and withdrawal rate), intervention characteristics (intervention content, frequency, duration, format and delivery mode), relevant outcomes assessed, how outcomes were measured, data collection time points, and the major findings. Data were extracted by one reviewer (BMHL) and the accuracy was verified by a second reviewer (RG). Disagreements on the extracted data were resolved through discussions between the reviewers.

### Study appraisal

The methodological quality of the included studies was evaluated using the revised Cochrane risk-of-bias tool for randomized trials (RoB 2) [30]. The appraisal tool aims to assess the included studies for the potential for bias arisen from (1) the randomization process, (2) deviations of trial procedures from the intervention as intended, (3) missing outcome data, (4) the process of outcome measurements, and (5) the selective reporting of study findings. Methodological quality of studies was rated as either low risk of bias, some concerns or high risk of bias. The quality assessments of the included studies were assessed independently by two reviewers (BMHL, RG), and disagreements in the ratings between the reviewers were resolved through discussions until a consensus was reached.

### Data synthesis and analysis

Data synthesis was performed using RevMan 5.4. The post-intervention means and standard deviations of outcome scores for each group were used for data synthesis. When outcomes were assessed using different scales or presented in other statistics, standardized mean difference and 95% confidence intervals were calculated to estimate the effect sizes. As a rule a thumb, the standardized mean difference (SMD) of 0.2, 0.5, and 0.8 were considered to be a small, medium, and large effect respectively [31]. The I^2^ test was used to measure heterogeneity. The random effects model was used for meta-analysis if I^2^ > 50.0%, which indicates the presence of significant heterogeneity [32]. The analysis was then graphically presented as a Forest plot. Publication bias were evaluated by visual inspection of funnel plots when at least 10 studies are measuring the same outcomes [33]. The significance level is set at *p* < 0.05.

### Certainty of the evidence

The certainty of evidence for the outcomes was evaluated using the Grading of Recommendations Assessment, Development and Evaluation (GRADE), facilitated by the Development and Evaluation profiler Guideline Development Tool (GRADEpro GDT). The assessment considered five key domains: risk of bias, inconsistency, indirectness, imprecision, and publication bias. Based on these criteria, the evidence was categorized into four levels of certainty: very low, low, moderate, and high [34].

## Results

### Search results

**Fig. 1:**
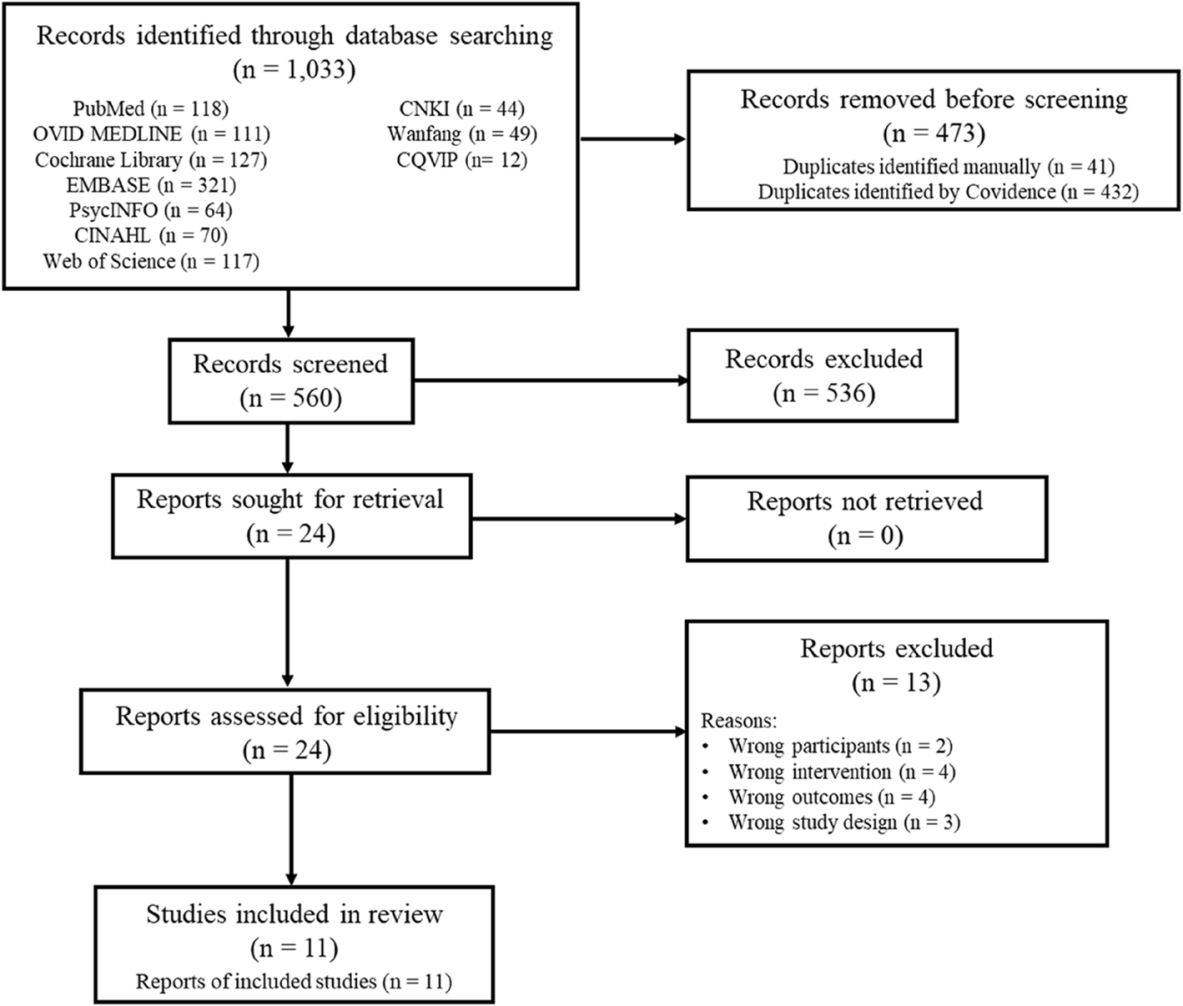
The PRISMA Diagram Depicting The Flow Of The Literature Search

A total of 1,033 citations were retrieved in the systematic research using the English databases. After the removal of duplicates (*n* = 473), the remaining 560 retrieved citations were screened for the titles and abstracts, of which 536 citations were excluded. The remaining 24 citations were assessed for their eligibility for inclusion through a full-text review. Of these, 13 articles were excluded due to wrong participants (*n* = 2), wrong outcomes (*n* = 4), wrong interventions (*n* = 4), and wrong study designs (*n* = 3). The remaining 11 articles underwent a critical appraisal of their methodological quality using RoB2 and were included in the critical synthesis. The PRISMA diagram depicting the flow of literature search and article selection is presented as Fig. 1.

### Included studies

Studies included in this review were published between 1997 and 2021. These studies have been conducted in Hong Kong, China [35], India [36], Mainland China [37, 38], New Zealand [39], Thailand [40], Turkey [41], the United Kingdom [42], and the United States [43–45]. The sample sizes ranged from 18 to 136. Six studies were conducted among older adults, either from community [35, 43, 44], hospital [38] or nursing homes [36, 41]. Other studies were among breast cancer survivors [40, 45], patients with traumatic brain injury [39, 42] and healthy female university students [37]. All studies involved self-esteem as one of their measured outcomes, except Okuyan et al. [41], where one of the studied outcomes was self-confidence perception instead. Details of the participants, interventions and outcome measurements of the included studies are presented in Supplementary Table 2.

### Results of Quality Appraisal

**Fig. 2:**
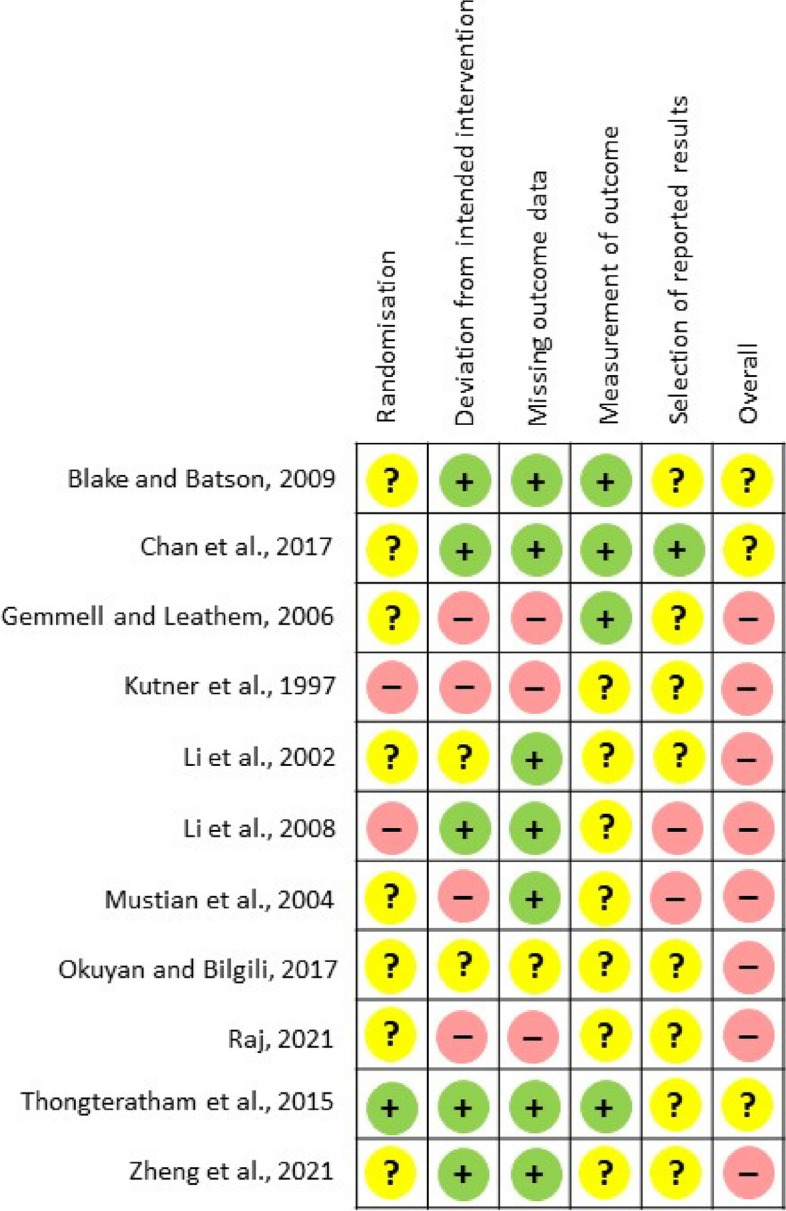
Critical Appraisal Of The Methodological Quality Of The Included Studies

The results of critical appraisal of the methodological quality of the included studies are summarized in Fig. 2. Overall, all studies were either rated as having “some concerns” (*n* = 3) or “having a high risk of bias” (*n* = 8). Regarding the randomization process, most studies (*n* = 10; 91.0%) were rated as “having some concerns” or “having high risk of bias” mainly due to the inadequate information provided on the procedure of randomization and/or the status of allocation concealment. Four studies (36.0%) were rated as having high risk of deviations from the intended interventions, primarily due to the inadequacy of information on the number of participants that were included in the analysis of data. The majority of the studies (*n* = 7) were at low risk of bias in the missing outcome data domain. Those at increased risk of bias for this domain were mainly caused by the significant proportion of participants who did not provide the data for analysis in the studies. For the domain of “measurement of the outcomes”, seven studies were rated as having some concerns. Such concerns were mainly related to the failure of the studies’ authors to state whether the outcome assessors were aware of the intervention received by the participants. The majority of the studies (*n* = 10) were rated as either having some concerns or a high risk of bias in selection of the reported results, as a pre-specified analysis plan was not made available, making it difficult to compare whether changes in methodologies used occurred between their proposed study plan and the actual procedures involved.

### Publication bias

**Fig. 3:**
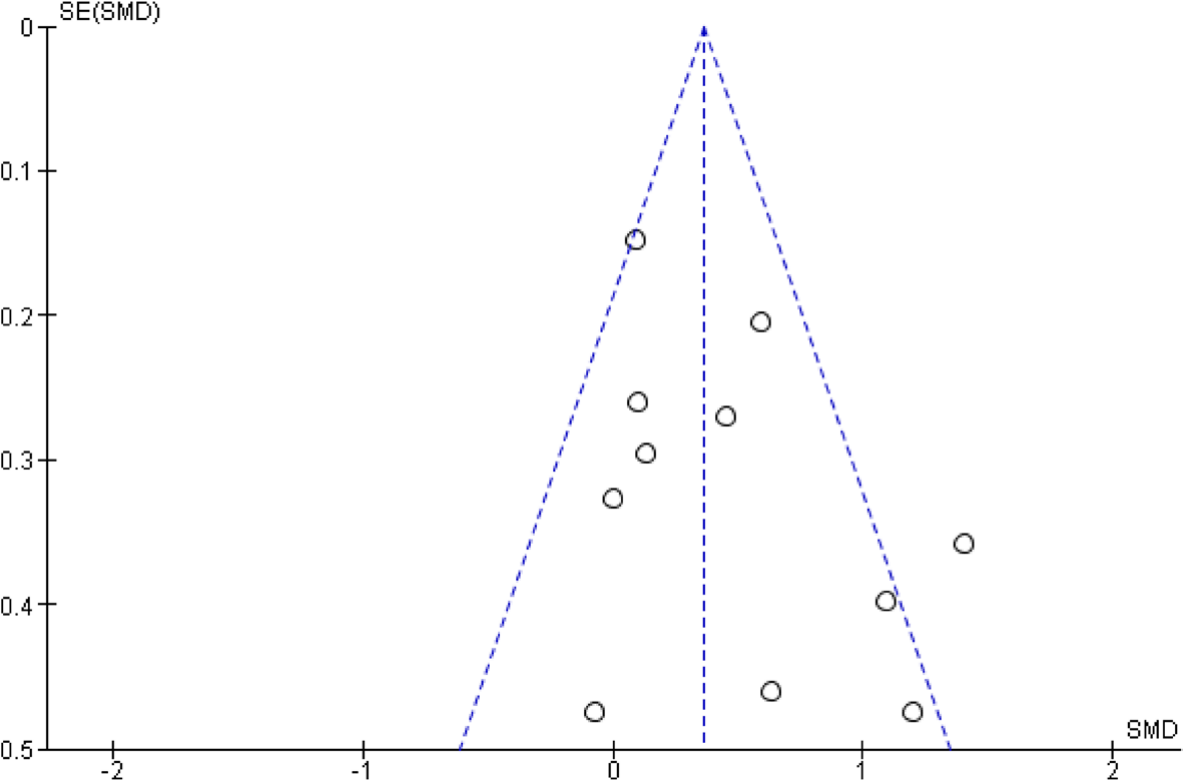
Funnel Plot Of Effects Of Tai Chi On Self-Esteem Compared With The Control Group Immediately Post-Intervention

The Funnel plot for the assessment of the publication bias is shown in Fig. 3. A reasonable inverted symmetry of the data points in the Funnel plot was observed, suggesting that the findings of our meta-analysis are not likely to be affected by publication bias.

### Intervention characteristics

Among the included studies, four adopted the Yang style Tai Chi as the Tai Chi style used in their interventions (*n* = 4) [41, 42, 44, 45]. The traditional Chen style Tai Chi was practiced in an intervention reported by one study [39]. However, most studies did not specify the Tai Chi styles used in their interventions [35–38, 40, 43]. There are also variations in the number of forms of Tai Chi practiced in the reported interventions among the included studies too. While one study involved the practice of 10 forms of Tai Chi [43], others involved the practice of more Tai Chi movements such as the 14 forms of Tai Chi [41], the 15 forms of Tai Chi [45], 18 forms of Tai Chi [35, 40, 42] and 24 forms of Tai Chi [37, 44]. One study practiced five forms of the traditional Chen style of Tai Chi [39]. Two studies did not state the number of Tai Chi forms practiced during the interventions [36, 38].

The duration of the Tai Chi session among the reported intervention ranges between 40 min and 60 min, with the frequency of the sessions ranging between once a week to five times per week. The range of the intervention duration was 4 weeks to 24 weeks.

### Intervention effects

Nine studies reported their findings using means and the associated standard deviations. One study reported the data using the median and interquartile range [42], and one study presented the F-statistics data from the repeated measures ANCOVA [45]. Given the heterogeneity in the use of statistical means for data reporting among the studies, all data were transformed to standardized mean difference and standard error for data synthesis. For the data reported by Blake and Batson [42], we had to assume that the overall distribution of their data did not deviate significantly from the normal distribution. Based on this assumption, the median would be treated as the mean, while the standard deviation is calculated by dividing the interquartile range by 1.35 [34]. For the data reported by Mustian et al. [45], the standardized mean difference was directly calculated using the Practical Meta-analysis Effect Size Calculator (https://www.campbellcollaboration.org/escalc/html/EffectSizeCalculator-SMD4.php) for use in the meta-analysis. The results were presented as standardized mean difference and its associated 95% confidence interval.

**Fig. 4:**
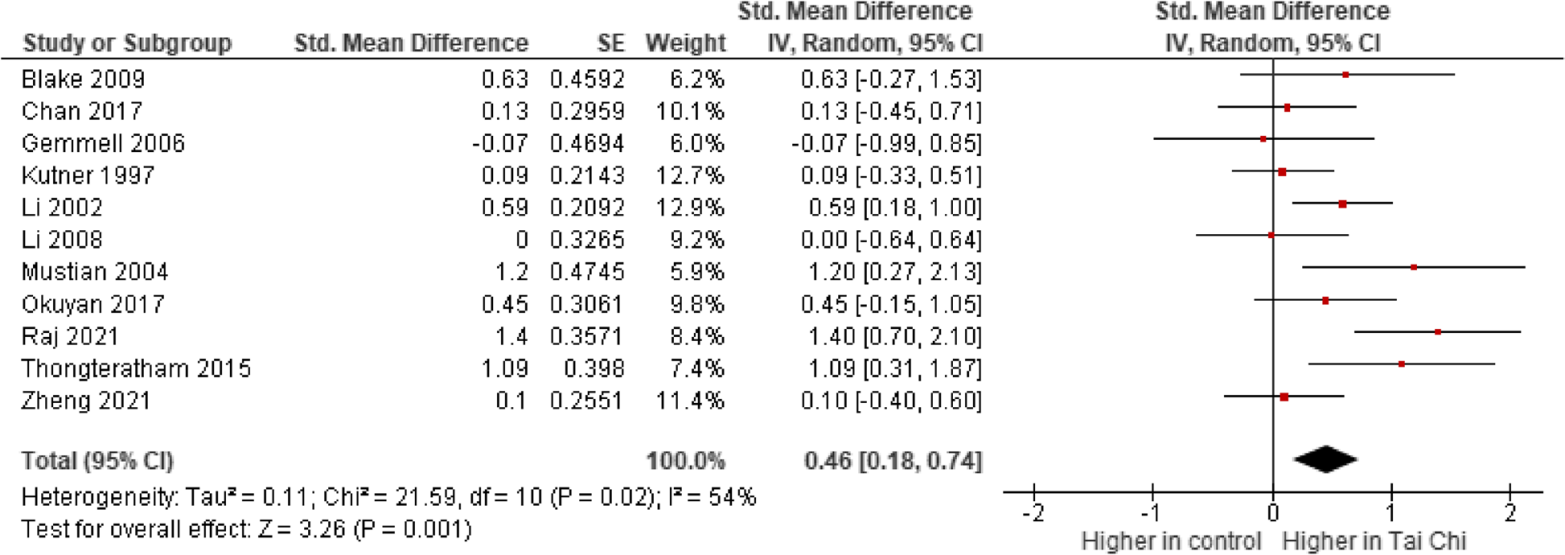
Forest Plot Showing The Effects Of Tai Chi Compared With The Control Group At Post-Intervention

The data from the 11 included studies were first pooled together. As shown in Fig. 4, the pooled estimates suggest that Tai Chi interventions can significantly improve self-esteem among the adult populations, as shown by the positive standardized mean difference in the post-intervention self-esteem score among the intervention participants, compared to controls (11 studies; Standardized mean difference: 0.46; 95% confidence interval: 0.18–0.74; *I*^*2*^ = 54.0%; *p =* 0.001). The level of certainty of the evidence was low because of the considerable risk of bias and different populations of studies (Table 1).

**Table 1:** Certainty of the evidence

Outcomes	№ of participants (studies) Follow-up	Certainty of the evidence (GRADE)	Relative effect (95% CI)	Anticipated absolute effects	
				Risk with [control group]	Risk difference with [Tai Chi]
Self-esteem	512 (11 RCTs)	⨁⨁◯◯ Low ^a, b^	-	-	SMD **0.46 SD higher** (0.18 higher to 0.74 higher)

Note: GRADE Working Group grades of evidence; CI: confidence interval; RCT: randomized controlled trial; SMD: standardized mean difference

High certainty: we are very confident that the true effect lies close to that of the estimate of the effect

Moderate certainty: we are moderately confident in the effect estimate: the true effect is likely to be close to the estimate of the effect, but there is a possibility that it is substantially different

Low certainty: our confidence in the effect estimate is limited: the true effect may be substantially different from the estimate of the effect

Very low certainty: we have very little confidence in the effect estimate: the true effect is likely to be substantially different from the estimate of effect

The risk in the intervention group (and its 95% confidence interval) is based on the assumed risk in the comparison group and the relative effect of the intervention (and its 95% CI)

a. “Some concerns” and “High” risk of bias

b. The sample includes various populations, e.g., older adults, breast cancer survivors, university students

Given that the estimation of the mean and standard deviation of the data from the Blake and Batson study [42] was based on the assumption of normal distribution of their data, the reported standardized mean difference of this study may therefore not truly reflect the real value. In light of this, a sensitivity analysis excluding this study was conducted. As shown in Supplementary Fig. 1, the exclusion of the Blake and Batson study from the analysis also resulted in the demonstration of a significantly positive effect of Tai Chi interventions on self-esteem among adults (10 studies; Standardized mean difference: 0.45; 95% confidence interval: 0.16–0.75; *I*^*2*^ = 58.0%; *p* = 0.003). We also conducted a sensitivity analysis by excluding one study [41] whose primary outcome was the perception of self-confidence, an outcome related to self-esteem. Such analysis consistently revealed a significantly positive effect of the Tai Chi intervention on improving self-esteem among adults (10 studies; Standardized mean difference: 0.47; 95% confidence interval: 0.16–0.77; *I*^*2*^ = 58.0%, *p* = 0.003) (Supplementary Fig. 2). However, the exclusion of these two studies did not lead to a substantial reduction in the I^2^ value for heterogeneity.

Finally, we conducted a subgroup analysis comparing the use of usual care and non-usual care among the control groups. Indeed, two studies involved the implementation of an established intervention among control participants, including an intervention involving social and leisure activities [42] and psychosocial support therapy [45], as opposed to the delivery of usual care or the use of wait-list control group among other included studies. We, therefore, tested for any subgroup differences in the outcomes between these two types of intervention-control comparisons. As shown in Supplementary Fig. 3, our subgroup analysis showed no significant differences in the outcome between the two types of comparisons (Chi^2^ = 1.96; *I*^*2*^ = 48.9%; *p* = 0.16), indicating that the included studies demonstrated a significant positive effect of Tai Chi intervention on self-esteem among adults across both types of control conditions. While the subgroup analysis did not fully account for the high level of heterogeneity observed, the findings suggest that Tai Chi interventions have a consistently positive effect on self-esteem in adult populations, regardless of control condition.

## Discussion

Our meta-analysis has presented a summary of the current evidence for the effectiveness of Tai Chi interventions in improving self-esteem among adults. Overall, we demonstrated a significant positive effect on both outcomes. The meta-analysis demonstrated a pooled effect size of 0.46 (95% confidence interval: 0.18–0.74), indicating that Tai Chi interventions exhibit a moderately positive effect on self-esteem. The sensitivity analysis involving the removal of one study using self-confidence perception as the outcome also yielded similar results, thereby further supporting the positive impact of Tai Chi interventions on self-esteem. Our review findings therefore provide further evidence for the prospect of the implementation of Tai Chi interventions to benefit individuals in need of improvement in self-esteem and self-confidence. However, given the high risk of bias in most of the included studies, caution is needed in interpreting these results and in recommending the widespread implementation of Tai Chi interventions until higher-quality evidence is available.

The multidimensional and hierarchical theory of self-esteem, Exercise and Self-Esteem Model (EXSEM), posits that physical activity acts on a parallel level with exercise self-efficacy through the mediation of subdomains of physical condition, attractive body, and strength, and domain levels of physical self-worth to enhance global self-esteem [46, 47]. Tai Chi, a moderate-intensity exercise, has been shown to positively affect physical health outcomes, including the improvement in balance and coordination that minimize the risk of falling [23, 48], and a reduction of risk of chronic diseases such as hypertension and cardiovascular disease [49–51]. As a low-impact exercise characterized by frequent semi-squat positions including concentric and eccentric muscle contractions, Tai Chi can enhance muscle strength [52]. Tai Chi is also deeply rooted in the cultural philosophies of Taoism, emphasizing tranquility of the mind and the integration of heaven and humankind [52]. Through promoting relaxation and reducing stress, Tai Chi fosters inner harmony, which can help balance the psychological effects [52]. Given its ability to improve physical health and physical self-worth, Tai Chi can be regarded as an ideal strategy for enhancing self-esteem.

In addition, Tai Chi has demonstrated benefits for psychological well-being, primarily through the alleviation of anxiety and depression [27], which in turn would lead to an improvement of quality of life [28]. Notably, Tai Chi was previously cited as a non-pharmacological approach for the treatment of mental disorders [53], further demonstrating its positive effect on psychological and mental health. In our review, we demonstrated that Tai Chi interventions have a significantly positive effect on self-esteem, an outcome that is related to psychological well-being. Indeed, self-esteem was found to be associated with perceived mental well-being [54], and low self-esteem was demonstrated to be a risk factor of anxiety and depression [55–57]. Therefore, a point that is interesting for exploration is whether Tai Chi interventions may exhibit their known effect on psychological well-being through its positive effect on self-esteem. In one of the included studies of our review [36], Tai Chi intervention was shown to be effective in both promoting self-esteem and reducing depression among depressed older adults, prompting a possibility that the two observable effects of the Tai Chi intervention could be related. Nevertheless, the study utilized a small sample size (*N* = 20) and a pretest-posttest study design, thereby making it difficult to assess the factors that cause the study participants to become less depressed after receiving the intervention. Further Tai Chi interventional studies involving a qualitative component are needed to confirm the hypothetical relationship between Tai Chi, self-esteem, and depression. In these studies, the participants’ views on why and how the Tai Chi intervention has improved their self-esteem and whether their reduction in depression is related to their improvement in self-esteem may be collected and analyzed, to provide further clues to the potential moderating effect of self-esteem on the positive effect of Tai Chi on depression.

It should be noted that there are variations in the number of forms of Tai Chi practiced in the reported interventions among the included studies, ranging from 10 forms to 24 forms of Tai Chi. Our data showed that these different forms of Tai Chi employed in the reported interventions resulted in similar positive effects on self-esteem. However, it is unclear whether higher number of forms of Tai Chi practiced in the intervention would result in better outcomes, and whether the practice of the Yang style of Tai Chi could yield more positive effects on self-esteem than the practice of the traditional Chen style of Tai Chi, given the limited number of studies included in this review. Recent evidence from a meta-analysis suggested that certain Tai Chi styles such as the 24-form Tai Chi have exhibited superior efficacy to other types in improving exercise capacity among older adults with Parkinson’s Diseases [58], suggesting that different level of effects may be observed with different Tai Chi styles. In view of this, further studies that compare the effects of different Tai Chi styles on self-esteem could be of value.

We acknowledge several limitations of our review. First, only articles published in English or Chinese were included in the review due to the language capability of the study team, and data obtained in relevant studies that are not published in these two languages were excluded from the analysis. This has limited the comprehensiveness of this review. Second, a significant number of the included studies were considered at high risk of overall bias in our critical appraisal of the methodological quality of the studies, and none of them were rated as low risk of bias. Third, five included studies focused on older adults, and three involved only female participants. Different intervention strategies may be required for various age groups and genders, considering the impact of demographic factors on outcomes. Further intervention strategies should be specifically tailored to address the needs of different demographic groups. Fourth, differences in participant characteristics, variations in the forms of Tai Chi interventions, and methodological heterogeneity among the included studies with high risks of bias might contribute to the higher heterogeneity observed in the results. Given the methodological limitations of the included studies that increase the risk of bias in their reported results, our review findings need to be interpreted with caution.

With Tai Chi interventions exhibiting effectiveness in improving self-esteem, an outcome highly related to mental health, Tai Chi practice should be promoted within the community, especially targeted to individuals with highlighted self-esteem needs, such as patients with body image affected (i.e., cancer patients), and older adults experiencing functional declining. Community organizations/institutions serving these aforementioned vulnerable groups may consider incorporating Tai Chi practice sessions into their regular health promotion service offered to local communities. Such Tai Chi sessions should be held as a group, a strategy that may promote a sense of togetherness and enhance social support [22]. These sessions may last for 40–60 min, with at least one session held per week, an intervention duration and frequency that are reported by most of the included studies. Caregivers working with these vulnerable groups may also form partnership with intervention deliverers, encouraging vulnerable individuals to participate in such group Tai Chi sessions. Through this, these individuals would thereby benefit from this non-pharmacological approach of psychological health promotion, at least in part through an improvement in their self-esteem.

## Conclusion

Overall, our review findings indicate a moderately positive effect of Tai Chi interventions on self-esteem among adult populations. Tai Chi was consistently shown to improve self-esteem despite the variations in the use of Tai Chi styles, and the duration and frequency of practice, among the included studies. Given these findings, Tai Chi could serve as a promising strategy for promoting self-esteem, particularly in communities with individuals in need of such interventions. However, due to the high risk of bias in many of the included studies, caution is advised before recommending the widespread implementation of Tai Chi until higher-quality evidence has become available. Further studies may consider involving the conduction of large-scale randomized controlled trials, exploring the effect of Tai Chi interventions on self-esteem with greater statistical power. Future studies may also be directed towards the examination of the views of Tai Chi intervention participants through qualitative interviews, exploring the major components of the intervention that have improved their self-esteem. The effect of different styles of Tai Chi on self-esteem may also be explored, determining the optimal intensity, duration, frequency and Tai Chi forms/styles that should be practiced in such interventions. Through such research, a recommended guideline of practice of Tai Chi interventions within the community can then be proposed, enabling such intervention to offer the greatest benefit to the intervention users and cost-effectiveness for implementation within the community.

## Supplementary Information

Supplementary Material 1.

Supplementary Material 2.

## Data Availability

Data is provided within the manuscript or supplementary information files.

## Abbreviations

CNKI: China National Knowledge Infrastructure
GRADE: Grading of Recommendations Assessment, Development and Evaluation
GRADEpro GDT: Development and Evaluation profiler Guideline Development Tool
RoB 2: risk-of-bias tool for randomized trials
SMD: standard mean deviation
