# Supplementary material for: The effect of the Tai Chi intervention on self-esteem and self-confidence perception in adult populations: a systematic review and meta-analysis
Source: BMC Nurs. 2025 Feb 14;24:174. doi: 10.1186/s12912-025-02792-9 (PMC11829346; doi:10.1186/s12912-025-02792-9)
Supplement: Supplementary file 2 — Supplementary Material 2. [file 12912_2025_2792_MOESM2_ESM.docx]

**Supplementary Table 1: The Search Strategy**

| **Database** | **Details** |
| --- | --- |
| Pubmed | Search: ((("Tai Ji"[Mesh]) OR ((((((((((((Tai Ji[Title/Abstract]) OR (Tai-ji[Title/Abstract])) OR (Taiji[Title/Abstract])) OR (Taijichuan[Title/Abstract])) OR (Tai ji chuan[Title/Abstract])) OR (Tai ji quan[Title/Abstract])) OR (Taijiquan[Title/Abstract])) OR (Tai chi[Title/Abstract])) OR (Taichi[Title/Abstract])) OR (Tai chi chuan[Title/Abstract])) OR (Tai chi quan[Title/Abstract])) OR (T'ai Chi[Title/Abstract]))) OR (((((((((((qigong[Title/Abstract]) OR (qi-gong[Title/Abstract])) OR (Qi Gong[Title/Abstract])) OR (Chi Kung[Title/Abstract])) OR (chi-kung[Title/Abstract])) OR (Ch’i Kung[Title/Abstract])) OR (Chi Chuang[Title/Abstract])) OR (Qi Chung[Title/Abstract])) OR (Chi Gong[Title/Abstract])) OR (taichi qigong[Title/Abstract])) OR ("Qigong"[Mesh]))) AND ((((((((((((*esteem[Title/Abstract]) OR (self-worth[Title/Abstract])) OR (self-concept[Title/Abstract])) OR (self-crit*[Title/Abstract])) OR (self-regard[Title/Abstract])) OR (self-confidence[Title/Abstract])) OR (self-perception[Title/Abstract])) OR (self-esteem[Title/Abstract])) OR (Self Concept[Title/Abstract])) OR (self-image[Title/Abstract])) OR (self-evaluation[Title/Abstract])) OR ("Self Concept"[Mesh])) |
| Embase | #1 exp Tai Chi/ or exp qigong/ or Tai Ji.mp.  or Tai-ji.mp. or Taiji.mp. or Taijichuan.mp. or Tai ji chuan.mp. or Tai ji quan.mp. or Taijiquan.mp. or Tai chi.mp. or Taichi.mp. or Tai chi chuan.mp. or Tai chi quan.mp. or T'ai Chi.mp.  #2 exp self esteem/ or exp self concept/ or *esteem/ or self-worth.mp. or self-concept.mp. or self-crit*.mp. or self-regard.mp. or self-confidence.mp. or self-perception.mp. or self-esteem.mp. or Self Concept.mp. or self-image.mp. or self-evaluation.mp.  #1 AND #2 |

**Supplementary table 2. The characteristics and relevant findings of the included studies.**

| **Author / year / Study ID** | **Participants** | **Intervention** | **Outcomes** | **Measurements** | **Major relevant findings** |
| --- | --- | --- | --- | --- | --- |
| Okuyan and Bilgili, 2017; Turkey | ***Overall sample***   - Older people living in a nursing home. - N = 56 - % women: Not specified - Mean age: Not specified   ***Intervention group***   - n = 26 - % women: Not specified - Mean age: Not specified - Withdrawal rate: 23% (n = 6) - Analyzed: n = 20   ***Control group***   - n = 30 - % women: Not specified - Mean age: Not specified - Withdrawal rate: 20% (n = 6) - Analyzed: n = 24 | ***Intervention group***  **Tai Chi Chuan intervention**   - Completion of a 12-week tai chi chuan program, using the Yang style of tai chi chuan. - The program was led by the first author of the study. - Sessions were held twice a week, each lasting 35-40 minutes. - Sessions involved 5 minutes of warm-up, 30 minutes of tai chi chuan practice and 5 minutes of cool-down exercise.   ***Control group***  **Usual care**   - Usual care exercises for 12 weeks, and did not receive the tai chi chuan program. | - Self-confidence perception | - Physical self-description questionnaire   ***Data collection timepoint***   - Baseline - Post-intervention | **Effect of intervention on self-confidence perception**  ***Intervention group***   - Baseline score: 3.65 ± 2.33 - Post-intervention score: 4.25 ± 1.74 - *p* = 0.000   ***Control group***   - Baseline score: 3.45 ± 1.76 - Post-intervention score: 3.45 ± 1.76 - *p* = 1.000 |
| Blake and Batson, 2009; United Kingdom | ***Overall sample***   - Adults with traumatic brain injury, recruited in a community setting at a community day center that provide support services to people with traumatic brain injury. - N = 20 - % women: 25% - Mean age: Not specified   ***Intervention group***   - n = 10 - % women: 10% - Mean age: 44.5 ± 10.5 years - Withdrawal rate: 0% (n = 0) - Analyzed: n = 10   ***Control group***   - n = 10 - % women: 40% - Mean age: 46.2 ± 11.3 years - Withdrawal rate: 10% (n = 1) - Analyzed: n = 10 | ***Intervention group***  **Tai chi qigong intervention**   - Completion of an 8-week tai chi qigong exercise program, supervised by a certified instructor of qigong with experience working with people with traumatic brain injury - Each tai chi qigong session took place once a week, each for 1 hour. - The program included a combination of qigong breathing and the Yang form of Tai Chi   ***Control group***  **Non-exercise social and leisure activities**   - Attendance to sessions of non-exercise social and leisure activities at the community day center. - Each session lasted 1 hour, held once a week for 8 weeks. - Activities included group discussions, table games, and attendance to drawing, reading and writing classes | - Self-esteem | - Physical self-description questionnaire   ***Data collection timepoint***   - Baseline - Post-intervention | **Effect of intervention on self-esteem**  ***Intervention group (median ± inter-quartile range)***   - Baseline score: 2.83 ± 0.96 - Post-intervention score: 3.44 ± 1.12 - Change in pretest-posttest score: 0.61 ± 0.16   ***Control group (median ± inter-quartile range)***   - Baseline score: 2.55 ± 1.12 - Post-intervention score: 2.88 ± 1.16 - Change in pretest-posttest score: 0.33 ± 0.04   There was no significant difference in the change of scores between baseline and post-intervention (*p* = 0.344) |
| Chan et al., 2017; Hong Kong, China | ***Overall sample***   - Individuals aged 60 or above who did not engage in any social activities. - N = 46 - % women: Not specified - Mean age: Not specified   ***Intervention group***   - n = 24 - % women: 75.0% - Mean age: 75.4 ± 5.9 years - Withdrawal rate: 16.7% (n = 4) - Analyzed: n = 24   ***Control group***   - n = 22 - % women: 77.3% - Mean age: 79.4 ± 8.5 years - Withdrawal rate: 31.8% (n = 7) - Analyzed: n = 22 | ***Intervention group***  **Tai chi qigong intervention**   - Participation in a tai chi qigong exercise class for a period of 3 months, with sessions held twice a week lasting for 60 minutes, led by a tai chi qigong instructor. - The intervention was held in an activity room at the district elderly community center, in small groups (<15 participants in each group). - The program involves the practice of 18 forms of tai chi qigong. After each session, participants were encouraged to self-practice the tai chi qigong for 30 minutes per day.   ***Control group***  **Usual care**   - Usual care involving home visits by social workers, at a frequency ranging between monthly and quarterly. - Participants received regular information of the community services available to them monthly. | - Self-esteem | - Rosenberg self-esteem scale   ***Data collection timepoint***   - Baseline (T0) - 3 months post-intervention (T1) - 6 months post-intervention (T2) | **Effect of intervention on self-esteem**  ***Intervention group***   - Score at T0: 19.2 ± 3.3 - Score at T1: 19.1 ± 3.7 - Score at T2: 20.8 ± 4.1   ***Control group***   - Score at T0: 17.8 ± 3.2 - Score at T1: 18.6 ± 4.1 - Score at T2: 19.0 ± 2.9   **Generalized estimating equation model for comparing changes of self-esteem rating from T0 between groups.**  ***Comparison between groups at T1***   - B = -0.71; 95% CI: -3.07 – 1.65; *p* = 0.557   ***Comparison between groups at T2***   - B = 0.64; 95% CI: -1.90 – 3.18; *p* = 0.621 |
| Gemmell and Leathem, 2006; New Zealand | ***Overall sample***   - Traumatic brain injury patients, recruited through a psychology clinic at a local university. - N = 18 - % women: 50% - Mean age: Not reported   ***Intervention group***   - n = Not reported - % women: Not reported - Mean age: Not reported - Withdrawal rate: Not reported - Analyzed: Not reported   ***Control group***   - n = Not reported - % women: Not reported - Mean age: Not reported - Withdrawal rate: Not reported - Analyzed: Not reported | ***Intervention group***  **Tai chi intervention**   - Participation in a tai chi course, with sessions held twice a week for a period of 6 weeks. Each session lasted 45 minutes. - The tai chi course was based on a traditional Chen style of tai chi. Participants were taught the breathing and stepping techniques and five forms from the 38-step frame. Sessions were instructed by instructors.   ***Control group***  **Wait-list control**   - Participants participated in the tai chi course after post-intervention data collection. | - Self-esteem | - Rosenberg self-esteem scale   ***Data collection timepoint***   - Baseline (T0) - 3 weeks post-intervention (T1) | **Effect of intervention on self-esteem**  ***Intervention group***   - Score at T0: 61.90 ± 22.68 - Score at T1: 56.66 ± 22.20   ***Control group***   - Score at T0: 65.00 ± 25.45 - Score at T1: 58.25 ± 18.88 - *p* > 0.05 |
| Kutner et al., 1997; United States | ***Overall sample***   - Community-living elderlies aged 70 or above. - N = 136 - % women: Not reported - Mean age: Not reported   ***Intervention group (Tai Chi group)***   - n = 72 - % women: Not reported - Mean age: Not reported - Withdrawal rate: Not reported - Analyzed: n = 51   ***Control group***   - n = 64 - % women: Not reported - Mean age: Not reported - Withdrawal rate: Not reported - Analyzed: n = 40 | ***Intervention group***  **Tai chi intervention**   - Participation in a tai chi course involving practice of 10 of the tai chi movements, supervised by an instructor in small groups of 12. They were also encouraged to practice these movements on their own, through the provision of pictures and written description of each form. - The course involved sessions held twice a week, for a period of 15 weeks. The duration of each session was not indicated.   ***Control group***  **Usual care**   - Participants joined in weekly sessions with the instructor to discuss topics that may be of interest to them, such as polypharmacy, diet and sleep patterns. - The duration of sessions were not specified, for a period of 15 weeks. | - Self-esteem | - Rosenberg self-esteem scale   ***Data collection timepoint***   - Baseline (T0) - Immediate post-intervention (T1) - 4 months post-intervention (T2) | **Effect of intervention on self-esteem**  ***Intervention group***   - Score at T0: 7.9 ± 2.3 - Score at T1: 8.2 ± 2.1 - Score at T2: 7.9 ± 2.1   ***Control group***   - Score at T0: 7.8 ± 2.3 - Score at T1: 8.0 ± 2.4 - Score at T2: 8.3 ± 2.3   *p* > 0.05 |
| Li et al., 2002; United States | ***Overall sample***   - Low-activity healthy older adults aged 65 or above, recruited within the community. - N = 98 - % women: Not reported - Mean age: 73.2 ± 4.9 years   ***Intervention group***  n = 53   - % women: 88% - Mean age: 72.8 ± 4.7 years - Withdrawal rate: 18% (n = 9) - Analyzed: n = 49   ***Control group***   - n = 45 - % women: 92% - Mean age: 72.7 ± 5.7 years - Withdrawal rate: 29% (n = 13) - Analyzed: n = 45 | ***Intervention group***  **Tai chi intervention**   - Participation in tai chi intervention that involved 60-minute sessions of tai chi held twice a week over a period of 6 months (24 weeks). - Participants practiced the classical Yang style (24 forms) of tai chi during the sessions. The sessions involved warm-up for 15 minutes, 30 minutes of tai chi practice and 15 minutes of cool-down. Participants were also encouraged to practice the tai chi at home.   ***Control group***  **Wait-list control**   - Participants carried out their normal routine activities during the study. Tai chi intervention was provided after post-intervention data collection. | - Global self-esteem - Domain-specific self-esteem | - Rosenberg self-esteem scale - Physical Self-perception Profile   ***Data collection timepoint***   - Baseline (T0) - 12 weeks after start of intervention (T1) - immediately post-intervention (24 weeks after start of intervention) (T2) | **Effect of intervention on self-esteem**  **Global self-esteem**  ***Intervention group***   - Score at T0: 32.31 ± 4.06 - Score at T1: 35.00 ± 3.82 - Score at T2: 35.23 ± 3.94   ***Control group***   - Score at T0: 31.07 ± 4.81 - Score at T1: 32.50 ± 4.55 - Score at T2: 32.72 ± 4.51   **Domain-specific self-esteem**  ***Physical self-worth scale***  ***Intervention group***   - Score at T0: 13.51 ± 4.03 - Score at T1: 15.14 ± 3.80 - Score at T2: 15.73 ± 3.61   ***Control group***   - Score at T0: 14.04 ± 3.03 - Score at T1: 13.65 ± 3.21 - Score at T2: 14.53 ± 3.10   ***Perceived attractive body***  ***Intervention group***   - Score at T0: 13.21 ± 3.95 - Score at T1: 14.54 ± 4.06 - Score at T2: 14.63 ± 3.81   ***Control group***   - Score at T0: 13.67 ± 3.58 - Score at T1: 14.06 ± 3.60 - Score at T2: 14.08 ± 3.62   ***Perceived physical strength***  ***Intervention group***   - Score at T0: 12.99 ± 3.67 - Score at T1: 14.19 ± 3.88 - Score at T2: 14.45 ± 3.99   ***Control group***   - Score at T0: 13.62 ± 3.47 - Score at T1: 13.24 ± 3.97 - Score at T2: 13.78 ± 2.84   ***Perceived physical conditions***  ***Intervention group***   - Score at T0: 13.42 ± 3.62 - Score at T1: 14.81 ± 3.85 - Score at T2: 14.95 ± 3.75   ***Control group***   - Score at T0: 13.31 ± 3.87 - Score at T1: 13.56 ± 4.01 - Score at T2: 14.11 ± 3.55 |
| Mustian et al., 2004; United States | ***Overall sample***   - Women breast cancer survivors, who were diagnosed with stage 0-IIIb breast cancer, recruited from a local cancer center of a local university. - N = 31 - % women: 100% - Mean age: 52 ± 9 years   ***Intervention group***  n = 17   - % women: 100 - Mean age: Not reported - Withdrawal rate: 35.3% (n = 6) - Analyzed: n = 11   ***Control group***   - n = 14 - % women: 100% - Mean age: Not reported - Withdrawal rate: 28.6% (n = 4) - Analyzed: n = 10 | ***Intervention group***  **Tai chi chuan intervention**   - Participation in a tai chi chuan program that lasted for 12 weeks, with 60-minute sessions held 3 times a week. - The tai chi chuan sessions were led by an experienced tai chi chuan instructor. The participants practiced the 15-move short form of Yang style of tai chi. - Each session began with a 10-minute warm-up session of stretching and basic chi kung, followed by 40 minutes of tai chi chuan practice and 10-minute exercise cool down period.   ***Control group***  **Psychosocial support therapy**   - Sessions were led by a graduate exercise psychology student (who was supervised by a counsellor), receiving education on behavioral coping strategies, peer support and group cohesion. - Sessions were held 3 times a week, lasting 60 minutes over a period of 12 weeks. | - Self-esteem | - Rosenberg self-esteem scale   ***Data collection timepoint***   - Baseline (T0) - 6 weeks after start of intervention (T1) - 12 weeks after start of intervention (T2) | **Effect of the intervention over time**  ***Repeated-measure ANCOVA demonstrated a significant main effect of time***   - The tai chi chuan intervention has a more positive effect on self-esteem of participants over time, compared with the control intervention   - F_1,19_ = 4.87, *p* = 0.04   ***A Trend for significant improvement in self-esteem from T0 to T2 among intervention participants, but not control participants***   - Intervention group: F = 3.36, *p* = 0.10 - Control group: F = 1.40, *p* = 0.27   ***Significant difference in self-esteem between groups at T2***   - F = 7.54, *p* = 0.01 |
| Raj, 2021; India | ***Overall sample***   - Depressed older adults recruited from a local old age home. - N = 40 - % women: Not reported - Mean age: Not reported   ***Intervention group***  n = 20   - % women: Not reported - Mean age: Not reported - Withdrawal rate: Not reported - Analyzed: Not reported   ***Control group***   - n = 20 - % women: Not reported - Mean age: Not reported - Withdrawal rate: Not reported - Analyzed: Not reported | ***Intervention group***  **Tai chi intervention**   - Participation in a tai chi class that lasted for 4 weeks. The frequency and duration of the sessions were not explicitly stated.   ***Control group***  **Usual care**   - Participants did not participate in the tai chi class | - Self-esteem | - Modified Rosenberg self-esteem scale   ***Data collection timepoint***   - Baseline (T0) - Post-intervention (30 days after start of the intervention (T1) | **Effect of intervention on self-esteem**  ***Intervention group***   - Score at T0: 11.65 (SD not reported) - Score at T1: 14.1 ± 3.1   ***Control group***   - Score at T0: Not reported - Score at T1: 10.5 ± 1.78   ***Significant pre-post increase of self-esteem (calculated t value>tabulated t value at 5% significance level)*** |
| Thongteratham et al., 2015: Thailand | ***Overall sample***   - Breast cancer patients having completed treatment for more than one year, who were diagnosed with stage 0-IIIb breast cancer, recruited at a breast clinic of a local university hospital. - N = 30 - % women: 100% - Mean age: Not reported   ***Intervention group***  n = 15   - % women: 100% - Mean age: Not reported - Withdrawal rate: 0% (n = 0) - Analyzed: n = 15   ***Control group***   - n = 15 - % women: 100% - Mean age: Not reported - Withdrawal rate: 0% (n = 0) - Analyzed: 15 | ***Intervention group***  **Tai chi qi gong intervention plus weekly telephone follow-up**   - Participation in a tai chi qi gong program involving 60-minute sessions of tai chi qi gong practice, held 3 times a week for a period of 12 weeks. - Each session involved 5 minutes of warm-up (muscle extension), 45-50 minutes of 18-form tai chi qi gong practice, and 5-10 minutes of cool-down. - Participants also received weekly telephone calls, conducted by the principal investigator, for monitoring and motivating the participants to practice tai chi qi gong at home 3 times a week.   ***Control group***  **Usual care**   - Participants were given a take-home leaflet and advices by the principal investigator. - Participants also received weekly telephone calls, conducted by the principal investigator, for monitoring and motivating the participants to do regular exercises at home 3 times a week | - Self-esteem | - Rosenberg self-esteem scale   ***Data collection timepoint***   - Baseline (T0) - 6 weeks after start of intervention (T1) - 12 weeks after start of intervention (T2) | **Effect of intervention on self-esteem**  ***Intervention group***   - Score at T0: 18.73 ± 2.60 - Score at T1: 19.60 ± 2.55 - Score at T2: 23.87 ± 3.54   ***Control group***   - Score at T0: 20.47 ± 2.56 - Score at T1: 20.73 ± 1.75 - Score at T2: 20.33 ± 2.74 |
| Li et al., 2008; China | ***Overall sample***   - Healthy women university students, with no regular exercise habits, recruited at a local university. - N = 38 - % women: 100% - Mean age: 20.35 ± 0.88 years   ***Intervention group***  n = 20   - % women: 100% - Mean age: Not reported - Withdrawal rate: 0% (n = 0) - Analyzed: n = 20   ***Control group***   - n = 18 - % women: 100% - Mean age: Not reported - Withdrawal rate: 0% (n = 0) - Analyzed: 18 | ***Intervention group***  **Tai chi chuan intervention**   - Participation in a tai chi chuan intervention that lasted for 8 weeks. Sessions were held 3 times a week, each lasting 60 minutes. - The sessions involved 10 minutes of warm-up, the practice of the 24-form tai chi chuan for 40 minutes, followed by 10 minutes of cool-down. Music was played in the background during the tai chi chuan sessions.   ***Control group***  **Usual care**   - Participation in exercise intervention and attendance to health talks on promotion of psychological well-being. - Sessions were also held 3 times a week for 60 minutes, over a period of 8 weeks. | - Self-esteem | - Profile of Mood States (POMS)   ***Data collection timepoint***   - Baseline (T0) - Immediately post-intervention (T1) | **Effect of intervention on self-esteem**  **POMS score for self-esteem**  ***Intervention group***  Score at T0: 7.28 ± 3.27  Score at T1: 9.36 ± 3.77  Within-group comparison*: p* = 0.000  ***Control group***  Score at T0: 8.28 ± 3.27  Score at T1: 9.36 ± 3.77  Within-group comparison*: p* = 0.092 |
| Zheng et al., 2021; China | ***Overall sample***   - Healthy older womens aged 60-75, with no regular exercise habits, recruited at a local hospital. - N = 60 - % women: 100% - Mean age: 65.32 ± 5.81 years   ***Intervention group***  n = 30   - % women: 100% - Mean age: Not reported - Withdrawal rate: 0% (n = 0) - Analyzed: n = 30   ***Control group***   - n = 30 - % women: 100% - Mean age: Not reported - Withdrawal rate: 0% (n = 0) - Analyzed: n = 30 | ***Intervention group***   - Participation in an intervention involving practicing simplified tai chi exercise. The intervention lasted for 12 weeks, with 40-minute sessions held 5 days a week. - The sessions included a 5-minute warm-up, 30 minutes of tai chi practice and 5 minutes of cool-down.   ***Control group***   - Usual care. Participants did not receive the intervention. | - Self-esteem | - Profile of Mood States (POMS)   ***Data collection timepoint***   - Baseline (T0) - Immediately post-intervention (T1) | **Effect of intervention on self-esteem**  **POMS score for self-esteem**  ***Intervention group***  Score at T0: 7.03 ± 2.42  Score at T1: 10.14 ± 3.63  Within-group comparison*: p* < 0.05  ***Control group***  Score at T0: 9.66 ± 2.42  Score at T1: 9.83 ± 2.51  Within-group comparison*: p* < 0.05  Between-group comparison: *p* < 0.05 |


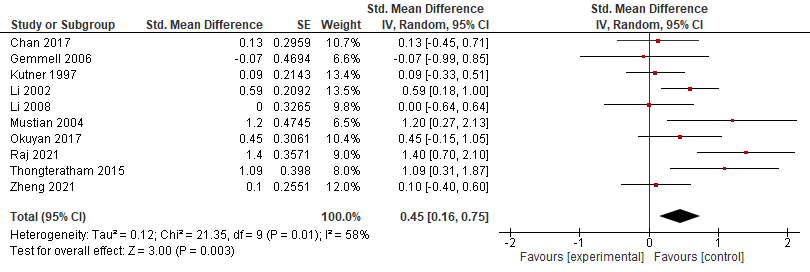


**Supplementary Figure 1:** Forest plot of a sensitivity analysis of the effects of Tai Chi compared with the control group at post-intervention (excluding the Blake and Batson 2009 study).


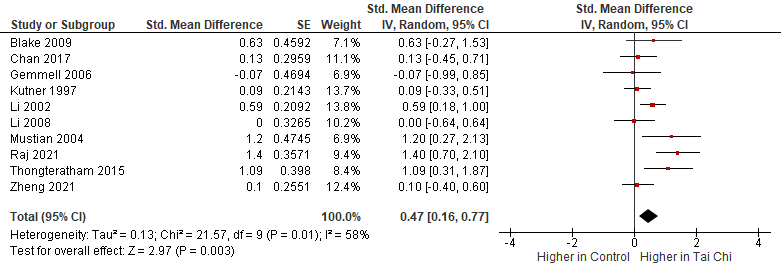


**Supplementary Figure 2:** Forest plot of a sensitivity analysis of the effects of Tai Chi compared with the control group at post-intervention (excluding the Okuyan and Bilgili 2017 study)


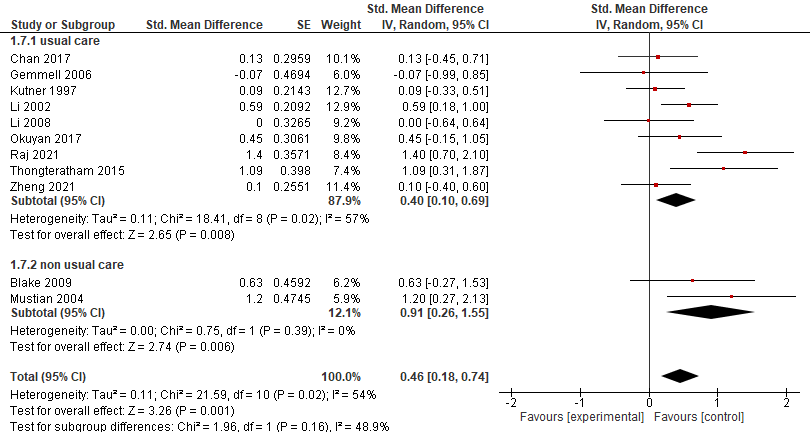


**Supplementary Figure 3:** Forest plot of the subgroup analysis comparing the difference in the effect of Tai Chi between control groups of usual care and those of non-usual care at post-intervention.
